# Deep reinforcement learning for automatic defocus correction using OCT image intensity

**DOI:** 10.1364/BOE.572077

**Published:** 2025-09-26

**Authors:** Guozheng Xu, Thomas J. Smart, Arman Athwal, Robert J. Zawadzki, Peter R. T. Munro, Marinko V. Sarunic

**Affiliations:** 1 Department of Medical Physics and Biomedical Engineering, University College London, London, WC1E 6BT, United Kingdom; 2 Institute of Ophthalmology, University College London, London, WC1E 6BT, United Kingdom; 3 UC Davis EyePod Small Animal Ocular Imaging Laboratory, University of California Davis, 4320 Tupper Hall, Davis, CA 95616, USA; 4 UC Davis Eye Center, Dept. of Ophthalmology & Vision Science, University of California Davis, Tschannen Eye Institute, 4860 Y St Suite 1E, Sacramento, CA 95817, USA; 5 Center for Human Ophthalmic Imaging Research (CHOIR), Dept. of Ophthalmology & Vision Science, University of California Davis, 4860 Y Street, Suite 2400, Sacramento, CA 95817, USA; 6 School of Engineering Science, Simon Fraser University, Burnaby, BC V5A 1S6, Canada

## Abstract

Optical coherence tomography (OCT) image stability often suffers during *in vivo* imaging of the retina due to axial motion of the subject’s head and changes in their visual focus. Ocular accommodation can actively adjust the focus, affecting the axial intensity distribution across the retinal cross-section and the lateral resolution of the target layers. Axial motion shifts the retinal image and affects *en face* visualization of retinal layers. We present an automated procedure for stabilization of axial motion and focus during OCT retinal image acquisition using deep reinforcement learning (DRL) for defocus correction. The correction process requires only B-scan images as inputs, making it suitable for real-time correction. *In silico* training and *in vivo* fine-tuning experiments have been conducted and presented to validate the performance of the correction procedure for retinal imaging.

## 1. Introduction

Optical coherence tomography (OCT) is a commonly used imaging modality for high axial resolution ophthalmic imaging [1]. Real-time *in vivo* imaging of the human retina often suffers from artifacts due to lateral and axial motion of the eye. Lateral motion eye tracking is an area of active research and development, with solutions integrated into commercial devices, and also in laboratory-grade instruments such as [2–7]. Additionally, the head movement of the subject and the accommodative micro-fluctuations of the intraocular lens are dominant sources of errors in the axial direction, which leads to the instability of the focal plane location within the axial field of view [8,9]. Focus changes affect the axial location of the focal plane and alter the axial intensity distribution of the cross-sectional OCT images. For *en face* visualization, defocus reduces the lateral resolution of the desired retinal layer. Axial motion affects the target layer selection and can make continuous *en face* visualization of a specific retinal layer challenging.

The stability of the focus is crucial for OCT imaging with instruments that have a high numerical aperture (NA) because the small focal waist at the sample rapidly diverges away from the focal plane. Changes in focus have significant effects on the axial intensity distribution in high-NA OCT B-scan images. Another factor that motivates the use of automated focus stabilization for high lateral resolution OCT is the application of adaptive optics (AO) [10]. AO has been applied to OCT retinal imaging to correct ocular aberrations and significantly increase the image quality [11]. As AO reduces the actual beam waist by eliminating aberrations, bringing it closer to diffraction-limited Gaussian width, the focus-changing effects become dominant, highlighting the need to be addressed.

High-resolution retinal imaging is commonly based on AO methods using direct measurement of ocular aberrations with wavefront sensors (WFS). Alternative approaches referred to as wavefront sensor-less (WFS-less) AO have also been demonstrated, which correct aberrations by optimizing image quality metrics through the reconstruction of the wavefront using varying modal wavefront coefficients [12–17]. Images used for optimization are often *en face* slices of the retina at a target depth. Under these scenarios, changes in focus during optimization alter the relative metric values used for optimization, resulting in the finding of suboptimal modal wavefront coefficient values for aberration correction. For WFS-less AO optimization based on *en face* images, axial motion also affects the target layer selection.

Dedicated hardware, allowing for the stabilization of focus and axial motion, has been widely used in OCT instruments. Tunable focus lenses are commercially available to adjust the focus of the optical system to a selected, specific target focal plane at the retina. OCT reference arms might have a fast adjustable axial length to control the relative axial position of the retinal image and to match variation in axial eye length. High-speed and small-scale axial pathlength changes can be achieved physically by a translation stage, electrically controlled motors, or voice coils [18–21]. The automation of these correction processes assists the system operators and increases the success rate of image acquisition sessions. Some automatic focus and axial position tracking methods have been developed for OCT to compensate for axial motion and focal accommodation. An approach to achieving continuous focus tracking and a depth-independent transverse resolution for real-time OCT imaging has been reported [22]. An automated hands-free focus tracking and z-tracking for point-of-care OCT that uses continuous gradient ascent optimization of focal position by searching for maximum B-scan intensity and a real-time detection of sample depth position in B-scans has been previously reported [23]. An automatic focus method using the interference fringes magnitudes of OCT has also been proposed [24].

Defocus and axial motion affect the depth-resolved images differently. Defocus alters the intensity distribution across the depth of the retina, while axial motion shifts the position of the retina layers. Images acquired with different focal planes will have distinct axial features in cross-sectional B-scan images since different retinal layers are being highlighted. The difference in image features could hamper the axial registration, especially when the images are single frames acquired for real-time interaction during acquisition. Axial shifts during the defocus optimization process may degrade the defocus correction performance because the target layer selected may change due to motion.

In this work, we present a systematic procedure of real-time non-iterative focus and axial motion correction for OCT retinal imaging, specifically with a single-step deep deterministic policy gradient (DDPG) method for defocus correction. DDPG is a self-learning agent that does not require labeled data and allows for interactive learning and hardware control [25–27]. The method works by continually stabilizing target B-scan frames during acquisition to a reference B-scan frame chosen by the user. Compared to iterative defocus optimization based on the sharpness of *en face* images, the B-scans provide higher-level information of the general axial intensity distribution, which can be mapped to focus differences non-iteratively by a deep neural network (DNN). In the procedure, the axial motion is corrected first through a pre-defined metric, and then the defocus is corrected by the single-step DDPG agent. One-dimensional lightweight convolutional neural network (CNN) structures are employed for the DDPG agent to process the A-scans averaged from the reference and target B-scans for fast fine-tuning and inference speed for real-time stabilization. The DDPG agent was initially trained and validated in a simulated environment. This was followed by fast fine-tuning and validations of performance and generalizability on *in vivo* retinal imaging sessions of human retina, proving the ability of the model to adapt to various retinal structures quickly for real-time axial motion and focus stabilization.

## 2. Method

### 2.1. OCT system

**Fig. 1.**
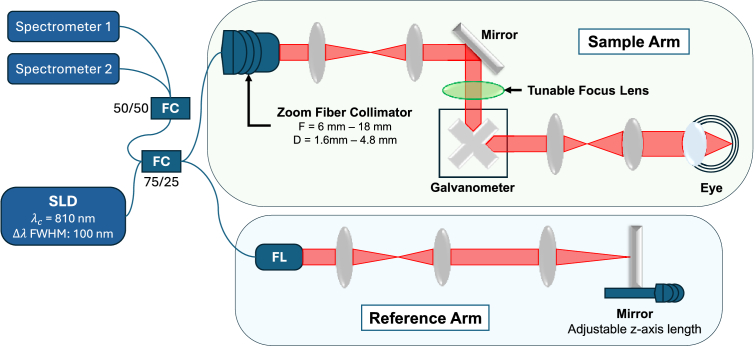
OCT system overview. FC: Fiber Coupler; FL: Fiber Launcher. The system utilizes a zoom fiber collimator with adjustable focal length from 6 mm to 18 mm, corresponding to a beam diameter at the eye from 1.6 mm to 4.8 mm. The superluminescent diode used has a central wavelength of 810 nm and a spectral width full width at half maximum (FWHM) of 100 nm.

We employed a custom-built dual-spectrometer spectral domain OCT (SD-OCT) for the demonstration of this method [28]. The schematic of the OCT system is shown in Fig. 1. The OCT is equipped with a zoom fiber collimator to adjust the beam diameter and, consequently, the NA for retinal imaging. The tunable NA range is approximately (0.04, 0.12). A Corning Varioptic Lens is used to change the axial position of the focal plane at the retina with a tunable range of (-5, 15) D. Data acquisition and OCT processing were performed using custom-developed software with GPU acceleration, allowing the A-scan rate of up to 400 kHz and real-time processing at up to 800 B-scan frames per second [29,30]. This software was previously demonstrated for real-time integration with AI-based layer segmentation [31].

### 2.2. Correction procedure

**Fig. 2.**
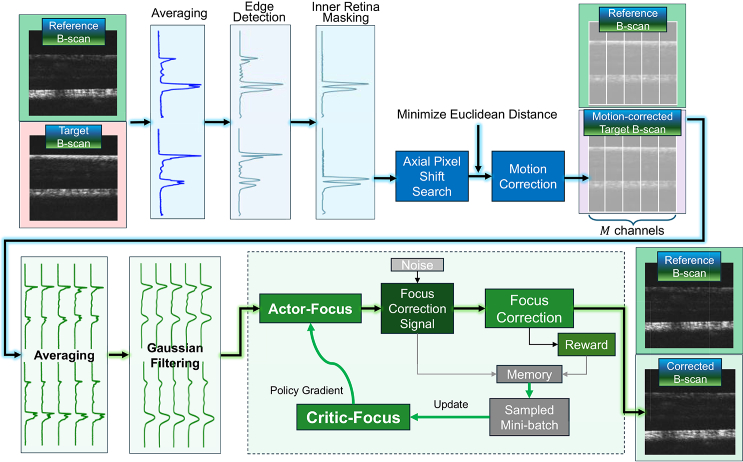
OCT B-scan axial motion and defocus correction procedure. The top row shows a block diagram of rapid axial motion correction; the bottom row shows a block diagram of AI-in-the-loop focus correction.

Figure 2 shows the OCT B-scan axial motion and defocus correction procedure. The reference B-scan is an arbitrarily chosen frame that the following frames are registered to, including both focus position depth and axial placement of the B-scan. The reference frame is a type of ‘promptable interface’ for the user to interactively select the appearance of the retina when the focus is placed at the desired image depth. During acquisition, the target B-scans are continuously acquired in real-time to be registered to the reference. The axial motion is corrected first based on post-processed A-scans averaged from the reference and target B-scans. The defocus is corrected by a DDPG agent in a single correction step based on the axial registration result.

#### 2.2.1. Rapid axial motion correction with different focal planes

The first step of the procedure is to correct axial motion. The axial motion correction uses the average A-scans from the reference and target B-scans. Next, we applied an edge detection map (EDM) to the A-scans by Gaussian filtering, first derivative calculation, and normalization of absolute values. The EDM highlights the inner and outer retinal layers that are crucial for retina axial registration, discards unnecessary information, and normalizes the reference and target A-scans. As the focus changes, the relative intensity between the inner and outer retinal layers is altered, forming two individual peaks in the A-scan and the EDM. The dual-peak property of the A-scans can be misleading, as traditional correlation methods might wrongly align the inner layer of the reference image with the outer layer of the target image or vice versa. To increase the robustness of the reward, we applied a peak detection to the EDM to find the rough location of the inner retina and the outer retina, and then masked out the inner retinal layers.

The metric for the axial motion correction agent is the negative Euclidean distance (ED) between the reference EDM $E_{r}$ and the target EDM $E_{t}$ with only outer retinal layer profiles:

(1) $$R_{m}=-ED(E_{r},E_{t})=-\sqrt{\frac{1}{N}\sum {\left(E_{r}^{n}-E_{t}^{n}\right)}^{2}}$$

where *N* is the number of pixels axially. By masking out the inner retina before comparing the similarity of the two EDMs, the agent learns to register the B-scans based on the closeness of the outer retinal layer. The axial motion by pixel $M_{p}$ is determined by the maximum $R_{m}$ found by shifting $E_{t}$ between a range of $N_{p}$ pixels. $N_{p}$ can be chosen by balancing the correction speed and the correction range.

(2) $$M_{p}=\underset{p}{\underbrace{argmax}}\left\{\left\{-ED\left(E_{r}\left(z\right),E_{t}\left(z-p\right)\right)\right\},p\in \left[-N_{p},N_{p}\right]\right\}$$

**Fig. 3.**
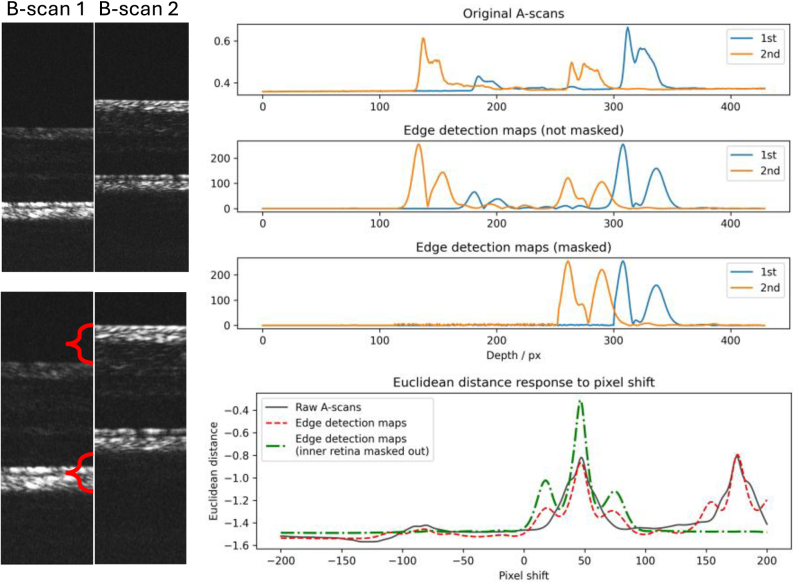
Comparison of metric responses to axial motion between data types used for axial motion correction. The left two B-scans are acquired under distinct focus positions, with the left focusing on the outer retina and the right on the inner. The bottom B-scans are zoomed for illustration of axial motion. The original A-scans, EDMs, and masked EDMs are displayed on the right side. The bottom right figure shows the Euclidean Distance response to pixel shift, where line types represent EDs calculated using each data type. Dual-peak profiles exist for Raw A-scans and EDMs, while only a singular peak at the axial shift location exists for EDMs with the inner retina masked out.

Figure 3 exhibits an example of axially registering two B-scans with distinct focus positions. The ED responses to pixel shift with all three data types of results peak at the same axial motion position. However, the raw A-scans and EDMs exhibit another peak at a different location, indicating imperfect registration and misregistration between the inner and outer retina. On the contrary, a distinguishable singular peak at the axial motion location exists for EDMs with the inner retina masked out.

#### 2.2.2. AI-in-the-loop focus correction

Focus correction uses the reference B-scan and the most recently acquired target B-scan after axial registration. The mapping between A-scan intensity distribution profiles and focus differences is learned by the DDPG agent. The initial axial registration enables a more accurate point-to-point comparison of A-scans for the focus correction DNN to learn the feature changes associated with defocus. To utilize sufficient information from the B-scan frames and account for retinal axial structure differences within OCT volumes, each B-scan is laterally divided into *M* sub-regions, where pixels are averaged axially to form an averaged A-scan. The A-scans are then Gaussian filtered for smoothing and noise reduction. A combination of 2 M averaged A-scans from the reference and the target B-scans is the input to the “actor” of the focus correction DDPG agent (Actor-Focus). The output of Actor-Focus is the focus correction signal, which is translated to a physical, tunable focus lens.

After focus correction, a new B-scan frame is acquired and compared to the reference, generating a similarity reward evaluating the focus correction effectiveness. If the agent is under training, an annealing Gaussian noise will be added to the focus correction signal to enhance exploration. The multi-channel averaged A-scans, focus correction signal, and the reward will be stored in memory (replay buffer) for the training of the agent. It is worth noting that since the correction process is a single-step decision and immediate rewards are acquired after correction, multiple noise profiles can be generated and applied to the focus correction signal to generate multiple rewards, further enhancing the exploration by the agent. Detailed explanations of the focus correction DDPG agent terminology and algorithm are provided in the Supplement 1.

The reward shaping for training the Actor-Focus is also based on ED. The basic process involves calculating the ED between two 1D arrays, with a normalization step to ensure that the comparison is not influenced by differences in the scales of OCT data. The normalization standardizes the 1D arrays by subtracting the mean and dividing by the standard deviation:

(3) $$A_{r,t}^{n}=\frac{A_{r,t}-mean\{\left[A_{r},A_{t}\right]\}}{std\{\left[A_{r},A_{t}\right]\}}$$

where $A_{r},A_{t}$ are the original reference and target 1D arrays, and $A_{r,t}^{n}$ is the normalized 1D array.

Although the initial registration from axial motion correction provides close point-to-point comparisons between B-scans with different focus positions, the Actor-Focus is expected to experience a certain level of axial motion to improve the robustness of focus correction under axial motion and to avoid overfitting of point-to-point depth-resolved features instead of focus-related intensity distribution features between A-scans. Therefore, we adopted a sliding-window ED that axially moves the target A-scan pixel-by-pixel and calculates the corresponding ED between the target and the reference A-scan at each location. The minimum ED acquired from the sliding-window process represents the best achievable similarity between the reference and the focus corrected frames:

(4) $$R_{f}=-\underset{b}{\min}\{ED(A_{r}^{n}(z),A_{t}^{n}(z-b)),b\in [-N_{b},N_{b}]\}$$

where $A_{r}^{n}$ and $A_{t}^{n}$ are the normalized average A-scans from the reference and target B-scans, $R_{f}$ is the reward for the focus correction agent, *b* is an integer sliding variable that shifts the B-scan by *b* pixels, and $N_{b}$ is a configurable limit for the sliding process, which can be fine-tuned to balance the tolerance to the axial motion and the reward calculation time. This sliding window ED reward shaping technique enables the Actor-Focus to predict defocus with a reasonable tolerance for axial motion, thereby increasing overall robustness.

### 2.3. DNN structures

The DNN structures for the actor and critic are both composed of 1-dimensional CNNs and fully connected layers. CNNs extract the features of the multi-channel A-scan input that determine the focus error, while the fully connected structures convert the feature maps to correction signals or rewards.

**Fig. 4.**
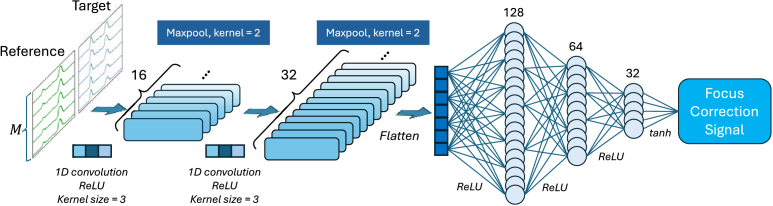
Actor network architecture for focus correction DDPG agent.

Figure 4 illustrates a CNN-based network for Actor-Focus, a one-dimensional convolutional neural network designed to generate a focus correction signal from the multi-channel observation. The architecture consists of two sequential blocks of 1D convolutional layers (each with a kernel size of three) and max-pooling operations (kernel size of two), which progressively increase the number of output channels (16 to 32) while reducing the temporal dimensionality. The final pooled feature maps are flattened, followed by a downstream fully connected chain of 128, 64, and 32 neurons with ReLU activation. The final focus correction signal output is generated with tanh activation after the last fully connected layer.

**Fig. 5.**
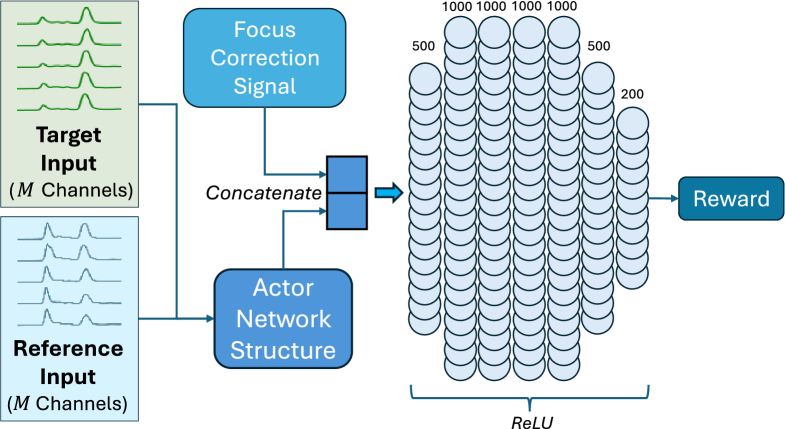
Critic network architecture for focus correction DDPG agent.

Figure 5 shows the critic network architecture for Critic-Focus that takes two sources of input. The first input is a single value output of the actor network block (shown in Fig. 4) fed with the multi-channel observation, and the second is the focus correction signal. Both inputs are then concatenated and fed into a deep feed-forward network comprising layers of 500, 1000, 1000, 1000, 1000, 500, and finally 200 neurons, each followed by a ReLU activation. The network’s final output is a single reward value evaluating the correction signal given the observation.

### 2.4. Simulation of OCT retinal image axial intensity distribution with defocus

The simulation of relative retinal axial intensity profile $I(z;z_{f})$ with different depth of focus of the eye $z_{f}$ employs a model considering the axial retina intensity reflectivity R(z) , amplitude attenuation by absorption and scattering A(z) , sensitivity roll-off H(z) of SD-OCT, confocal point spread function (CPSF) $T(z-z_{f})$ , and noise N(z) [32]:

(5) $$I(z;z_{f})=R(z)T(z-z_{f})A(z)H(z)+N(z).$$

The CPSF is derived from the beam divergence correction factor and yields the following expression for a Gaussian beam and diffuse reflection of the retina:

(6) $$T(z-z_{f})=\frac{1}{{\left(\frac{z-z_{f}}{2nz_{R}}\right)}^{2}+1},$$

where n is the refractive index within the retina, and $z_{R}$ is the Rayleigh length of the Gaussian beam incident on the sample [33]. Since the system does not have AO for ocular aberration correction, the Rayleigh length $z_{R}$ should incorporate a reasonable beam quality factor $M^{2}$ to simulate the reduced beam quality due to ocular aberration.

The attenuation by absorption and scattering can be expressed by an exponential decaying model as:

(7) $$A(z)=\mu_{B,NA}\exp(-2\mu_{OCT}z),$$

where $\mu_{B,NA}$ is a power-backscattering coefficient [34].

The expression for sensitivity roll-off H(z) can be expressed by:

(8) $$H(z)=\frac{\sin^{2}(\pi z/2z_{RD})}{{\left(\pi z/2z_{RD}\right)}^{2}}\exp\left[-\frac{\pi^{2}\omega^{2}}{8\ln2}{\left(\frac{z}{z_{RD}}\right)}^{2}\right],$$

where $z_{RD}=\lambda^{2}/(4\Delta \lambda )$ is the maximum ranging depth where Δλ is the wavelength spacing between pixels, and ω=δλ/Δλ where δλ is the spectrometer’s spectral resolution (FWHM) [35,36].

In simulation sessions, an OCT B-scan of the target retina at the target location is acquired under low NA with a large depth of field (DOF). This B-scan serves as the primary axial retina intensity reflectivity R(z) , preserving the relative reflectivity relationship between layers with fewer effects from the beam divergence. The effects of focal depth $z_{f}$ changes on the axial intensity profile were simulated by applying different $z_{f}$ values to the CPSF $T(z-z_{f})$ and calculating $I(z;z_{f})$ .

Nevertheless, accurate simulation of intensity requires other factors such as fiber coupling efficiency, input beam amplitude, etc. In this paper, we center on the relative intensity distribution at different focus positions and omit these depth-independent factors.

## 3. Results

### 3.1. In silico model training & evaluation

The simulation environment was developed under the system settings described in Section 2.1 and physics in Section 2.4. The zoom fiber collimator focal length was set to 12 mm, corresponding to an NA of about 0.1 in the eye. The training data were acquired from a subject with measured eye axial length and retinal layer thickness. A series of benchmark B-scan frames acquired at a specific focus position was chosen for the balanced illumination at the inner and outer retinal layers to perform defocus and axial motion simulation completely online and match the real-time defocus effects on the B-scan axial intensity redistribution.

The simulated random defocus was uniformly distributed between (-0.6, 0.6) D and the random axial movement between (-200, 200) µm. The amount of uniform distribution of both errors was sufficient for covering the axial motion and accommodative micro-fluctuation of a normal human subject whose head is fixed by a chin rest and eye focused on a fixation target placed at infinity. In reality, the distribution of focus errors should follow a Gaussian distribution, but for model training, we applied the large-bound uniform distribution for the agent to experience more severe defocus conditions. Also, to increase the variance of the original location of the retina in the B-scan images, we digitally applied a random initial motion to the images that moved the retina axially within a range of 200 µm. Similarly, the reference B-scan focus was randomly chosen and had a variation of 0.5 D to cover the entire retina.

The axial motion was corrected first, providing a more accurate side-by-side comparison of defocus effects for the focus correction agent. The mean error in focus correction at the end of training was 0.015 D. The device used was a PC with an Intel i5-13600 K CPU and an Nvidia RTX 4080 Super GPU. The average execution time for the axial motion correction and defocus correction was approximately 1 ms and 1.5 ms, respectively.

**Fig. 6.**
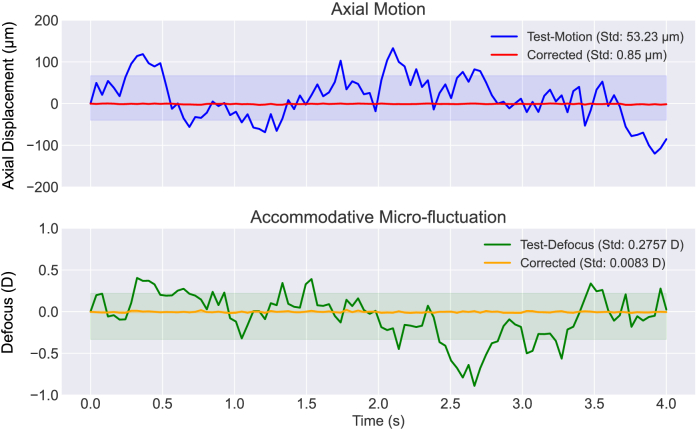
Example correction session of simulated axial motion and accommodative micro-fluctuation. A comparison between the test conditions (test motion and test defocus) and residual axial motion and defocus error after correction. The top panel displays axial motion, where the Test-Motion trajectory (blue) shows significant fluctuations (standard deviation: 53.2 µm), which the axial motion correction reduced to a standard deviation of 0.85 µm (red). Similarly, the bottom panel depicts accommodative micro-fluctuations, with the real-time data (green) exhibiting a standard deviation of 0.27 D, reduced to 0.008 D (orange). The shaded regions indicate the respective ranges of variability.

**Fig. 7.**
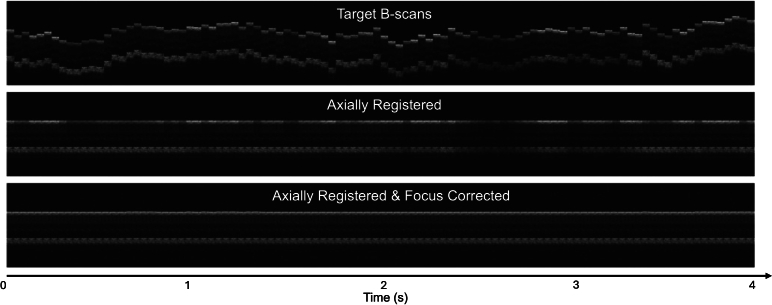
Kymograph visualization of an example simulated correction session. The first B-scan of the target images was used as the reference. The middle row demonstrates the results of image axial stabilization only, with axial intensity distribution variations across time. The bottom row reveals a uniform appearance of the motion and defocus corrected B-scans.

We demonstrated the model performance under a simulated OCT imaging session, where the random axial motion and accommodative micro-fluctuation were modeled by the Ornstein-Uhlenbeck (OU) process, a stochastic differential equation that effectively models continuous, random fluctuations with a tendency to revert to a fixed equilibrium position [37]. The simulated imaging time was 4 seconds, where the axial motion and focus changed every 40 ms, resulting in 100 test samples in total. Since the total prediction time of the correction procedure was much shorter, we simply treated the real-time correction as individual correction tests only with continuous axial motion and focus error. Figure 6 shows an example simulated correction session of axial motion and accommodative micro-fluctuation. Figure 7 is the Kymograph view of the correction session where the consecutive B-scans are projected laterally and displayed in a row as time-varying A-scans. The focus and axial motion errors before and after correction correspond to the numerical results in Fig. 6.

### 3.2. In vivo validation

**Fig. 8.**
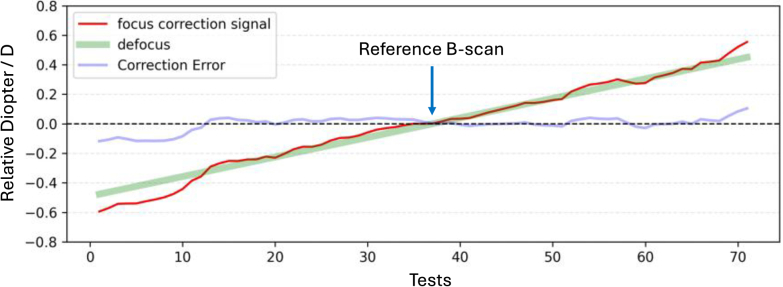
*In vivo* defocus correction test with an agent trained *in silico.* This figure shows the effectiveness of the predictions of defocus between reference and target B-scan frames by the focus correction agent trained *in silico*. 71 linearly changing defocus values (green line) were applied to the system, and corresponding target frames were acquired to be compared to the reference for focus signal predictions (red curve). A single focus correction signal output is collected from the Actor-Focus at each test. The correction error (blue curve) is the difference between the predicted and applied defocus values. The horizontal axis shows the test indices (cardinal numbers), and the vertical axis shows the diopter values relative to that of the reference frame.

For *in vivo* validation, we first examined the performance of the model trained by synthetic data directly applied to *in vivo* imaging sessions. The performance was acceptable within a small defocus range, but the prediction error increased quickly with a larger amount of defocus (Fig. 8). For optimal *in vivo* performance, model fine-tuning with *in vivo* data is required.

Challenges exist for *in vivo* training of agents to perform focus stabilization. The focus correction agent needs immediate focus correction effectiveness reward feedback after each correction attempt in one episode, meaning that continuous interaction with the optical system and engagement of the subject are required. Continuous imaging of the eye causes eye fatigue, which is clinically unideal for the subjects being imaged. The intermittent blinking of the eye also ruins image acquisition and further slows down the training. To address the challenges, we employed an A-scan focus interpolation method to reduce the amount of data acquisition required while providing sufficient *in vivo* data for fine-tuning of the model.

#### 3.2.1. Direct in vivo application of in silico focus correction model

We conducted an *in vivo* stabilization experiment on the OCT system (Fig. 1) on the same subject whose data were used for *in silico* training, but imaged a different retinal eccentricity.

The reference B-scan was acquired with the focus on the outer retina. The target B-scan frames were acquired in a swept-focus manner, achieved by the tunable focus lens that sampled the diopter values around the reference focus within (-0.5, 0.5) D. Random movement was intentionally generated during the acquisition. This emulates the eye accommodation with a structured defocus change for model evaluation and the axial motion. The correction accuracy of defocus was evaluated by the difference between the recorded swept-focus values and the predicted defocus values by the agent.

Figure 8 shows the diopter change of the tunable lens and the focus correction signal predicted by the focus correction agent. The agent successfully corrected the defocus with an average error of 0.02 D when the defocus was within (-0.3, 0.3) D, proving that the simulation successfully identified the core informational changes in B-scans associated with defocus. Nevertheless, the agent’s correction accuracy decreased to 0.035 D overall as the defocus increased beyond 0.4 D. Fluctuations in correction errors can be caused by imperfect simulation or minor eye accommodation during testing.

#### 3.2.2. Model fine-tuning with A-scan focus interpolation

The direct application of models trained in simulation on unseen retina structures proves the extrapolation ability of the network design; however, the networks are not designed to stabilize all retinal structures on all subjects, which requires a large database for training. Instead, the lightweight design allows for swift fine-tuning for the specific target retinal locations.

As discussed above, *in vivo* fine-tuning of the focus correction agent can be time-consuming and demanding for the subject to stabilize. We adopted an A-scan focus interpolation method to generate A-scans at different focus positions, reducing the amount of data acquisition and migrating the training process online without imaging subject involvement. The details for data acquisition, Data processing, and focus interpolation are described below.

1. Data acquisition. We sparsely sampled the focus position and recorded the corresponding B-scan images. For a focus range of (-1, 1) D, 21 samples with a 0.1 D interval were experimentally sufficient for accurate interpolation. The sampling frequency of focus at different locations was optimized to sample more data when the focal plane transits between retinal layers and sample less data at out-of-focus positions. With our system settings, batches of B-scans were acquired and processed in 10-20 ms, depending on the batch size to be processed, meaning that the acquisition of 20 samples is under 0.5 s. Under this timeframe, we assumed that the focus and axial position stability of the B-scans could easily be maintained with minor effort of cooperation from the subjects.
2. Data processing. B-scans were registered axially using the motion correction algorithm (Fig. 2) to correct any residual axial motion within the imaging window. The registered B-scans were then processed by lateral channel division, averaging, and Gaussian filtering (Fig. 2). The Gaussian filtering is critical since it removes the bumpy and noisy features in the A-scans while keeping the general intensity distribution map that determines the focus position.
3. Focus interpolation. We combined the processed A-scans of different focus positions into a 2D array where the first axis is the focus and the second is the depth of the retina. For each pixel of depth, we calculated the A-scan intensity values corresponding to interpolated focus values.

After interpolation, the data have a much higher focus resolution that can be used for model fine-tuning, where the *in vivo* A-scans at different focus positions can be substituted by inferences in the interpolated A-scan focus map. The fine-tuning sessions take about 2-5 minutes on a desktop computer with an Intel i5-13600 K CPU and an Nvidia RTX 4080 Super GPU, depending on the hardware and the performance of the model before fine-tuning on unseen data.

**Fig. 9.**
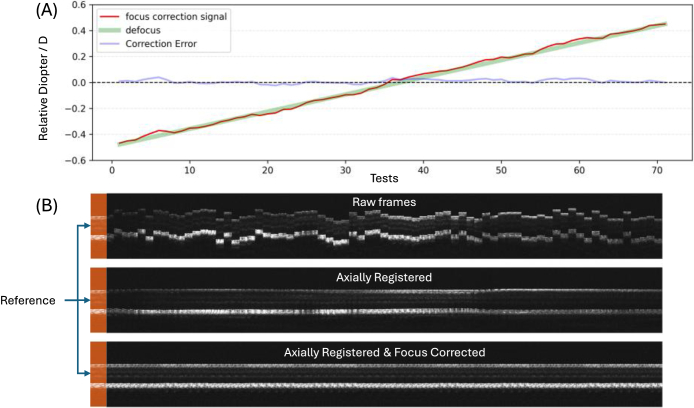
*In vivo* defocus & axial motion correction test with fine-tuned agent. Panel A shows the effectiveness of the predictions by the focus correction agent with different defocus values between the reference and target frames. 71 linearly changing defocus values (green line) were applied to the system and corresponding target frames were acquired to be compared to the reference for focus signal predictions (red curve). A single focus correction signal output is collected from the Actor-Focus at each test. The correction error (blue curve) is the difference between the predicted and applied defocus values. The horizontal axis shows the test indices (cardinal numbers), and the vertical axis shows the diopter values relative to that of the reference frame. Panel B is a visualization of 71 B-scan frames (projected laterally for visualization) corresponding to the line graph, demonstrating the effects from both the axial motion and defocus errors (raw frames), the effects of axial motion correction (axially registered), and the correction of both errors (axially registered and focus corrected).

Figure 9 shows the prediction accuracy after fine-tuning the original model trained on simulated data for an additional 3 minutes on real A-scans with focus interpolation. The average correction error with defocus ranging from (-0.5, 0.5) D is 0.015 D. Compared to the direct application of the model trained *in silico*, the fine-tuned version performs consistently within a 20-40% larger correction range and lower correction error.

**Fig. 10.**
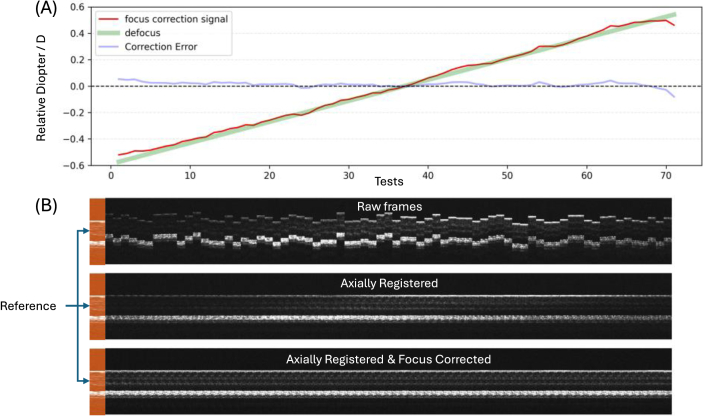
*In vivo* defocus & axial motion correction test with fine-tuned agent at unseen retina location. Panel A: Statistical visualization of focus correction effectiveness. Panel B: B-scan flow visualization of axial motion & focus correction effectiveness. The figure structure is the same as described in Fig. 9.

To further demonstrate that the focus correction agent learns the key features associated with focus changes instead of region-specific details, we tested the same fine-tuned model on a completely different eccentricity of the retina. The average focus correction error is 0.02 D, details shown in Fig. 10. Animated visualizations of the B-scans and averaged A-scans are provided in the Supplement 1 as Visualization 1 and Visualization 2, respectively.

## 4. Discussion

In this paper, we presented a real-time defocus and axial motion correction procedure for OCT retinal imaging, specifically using deep deterministic policy gradient for defocus correction. The axial intensity distribution profile is used for the determination of both axial motion and focus shift. The effectiveness of the approach was demonstrated with a simulation based on an SD-OCT system that incorporates the physical parameters that determine the A-scan retinal reflectivity profile response to focus position. The successful stabilization of the simulated axial motion and estimation of the position of the focal plane prove that the A-scan retinal intensity profile is sufficient for determining and correcting the focus and axial motion.

We adopted a pixel-shift optimization of Euclidean Distance for real-time axial motion correction between B-scans. An inner retinal layer masking technique was used to make the correction robust to focus changes that alternate between inner and outer retinal layers. For foveal regions with only bright structures at the outer retina, the number of detected high intensity peaks is less than 2, and no mask is applied. The axial motion correction method we used could be replaced with various digital image registration methods with higher accuracy (subpixel resolution) if they have sufficient speed for real-time OCT B-scan acquisition and operate against varying axial intensity distributions effectively.

The simulation of retinal axial intensity distribution changes due to defocus only addresses the features that affect the retinal A-scan intensity distribution but does not provide accurate full-wave analysis of the system. The focus correction agent trained in simulation was capable of predicting focus errors for *in vivo* data, but it did not fully capture the retinal A-scan features associated with defocus and required fine-tuning on real data. Nevertheless, the initial purpose of the simulation is for the early demonstration of the hypothesis that retinal axial intensity profiles can be used for focus prediction. In addition, with more robust simulation environments, models can be trained to incorporate more diverse retinal structures, which can potentially work for various system settings and retinal structures and circumvent real-time fine-tuning.

We considered that it is very challenging to train a single large model that identifies the image features from different retina locations, different modalities of OCT, and different subjects with healthy or diseased retinas. To make the method easily reproducible on various OCT systems and subjects, instead of training a single large foundation model, we employed lightweight one-dimensional CNNs for the DDPG agent to have fast adaptive learning capability. The lightweight networks are proven to be effective in identifying the mapping between OCT A-scan intensity signals and defocus. With the fast real data acquisition and interpolation technique for *in vivo* transfer learning, transfer learning or fine-tuning for a new device or new subject is feasible and easy to adopt. Nevertheless, an inclusive model with deeper layers and more complex structures can be trained to cover a wide range of retina structures through simulation or accumulated datasets of B-scans with labelled focus positions.

We have demonstrated the results of using OCT image intensity for focus correction on healthy retinas as a proof of concept. The limitations preventing validation on diseased retinas at the current stage of research are threefold. First, recordings of focus-shifted OCT data on a reasonably large cohort of diseased retinas, which are needed for model training and validation, are not available. Second, accurate simulation of diseased retinas is more challenging than healthy retinas since the layered structures can be altered. Finally, diseased retinas have more irregular shapes that may require more complicated network structures to identify the focus-related features. Nevertheless, the method still has strong potential to work in early disease detection scenarios where the layered structures of the retina are still mostly preserved.

The correction procedure described in this report demonstrates that axial profiles can be used to identify focus-related features in OCT B-scans of the retina. It also demonstrates the capability and generalizability of the network designs for stabilization of focus and exhibits fast fine-tuning speed and accuracy on target retinal locations. Further work is expected to reduce the time required for fine-tuning, such as applying automatic early termination criteria, and developing a fully automatic fine-tuning process for seamless and clinically friendly *in vivo* applications. Eventually, this tool has the potential to help stabilize the focus and axial motion for WFS-less AO optimization towards faster and more robust high-resolution retinal imaging with AO-OCT.

## 5. Conclusions

This paper presents an axial motion and defocus correction procedure for OCT image acquisition, specifically using deep deterministic policy gradient for defocus correction. The key feature providing information for axial motion and defocus correction is the axial intensity distribution of the retinal cross-sectional OCT image. A model initially trained *in silico* effectively identified the focus change between frames in a simulated imaging session and an *in vivo* imaging session. The novel A-scan focus interpolation method permitted fast *in vivo* data acquisition and model fine-tuning. The result proves the feasibility of *in vivo* focus stabilization through axial features of the retinal OCT data capable of achieving an average correction accuracy of 0.02 diopters.

## Supplemental information

Supplement 1 Supplemental document of explanations of focus correction DDPG agent terminology and algorithm https://doi.org/10.6084/m9.figshare.30182878

Visualization 1 The test conditions are the same as Fig. 9 and Fig. 10 in the main article. Raw frames are acquired with random axial motion and incremental defocus changes (from -0.5 D to 0.5 D) through a tunable focus device. Motion corrected frames exhibit the in https://doi.org/10.6084/m9.figshare.29948942

Visualization 2 The test conditions are the same as Fig. 9 and Fig. 10 in the main article. Raw frames are acquired with random axial motion and incremental defocus changes (from -0.5 D to 0.5 D) through a tunable focus device. Motion corrected frames exhibit the in https://doi.org/10.6084/m9.figshare.29948945

## Data Availability

Data used for this publication is not available.

## Supplemental document

See Supplement 1 for supporting content.
