# Supplementary material for: Deep reinforcement learning for automatic defocus correction using OCT image intensity
Source: Biomed Opt Express. 2025 Sep 26;16(10):4175–89. doi: 10.1364/BOE.572077 (PMC12532345; doi:10.1364/BOE.572077)
Supplement: Supplementary file 1 [file boe-16-10-4175-s001.pdf]

Supplemental Document

Biomedical Optics EXPRESS

Deep reinforcement learning for automatic defocus correction using OCT image intensity: supplement

GUOZHENG XU,[1,*] THOMAS J. SMART,[2] ARMAN ATHWAL,[1] ROBERT J. ZAWADZKI,[3,4,5] PETER R. T. MUNRO,[1] AND MARINKO V. SARUNIC[1,2,6]

[1]*Department of Medical Physics and Biomedical Engineering, University College London, London, WC1E 6BT, United Kingdom*
[2]*Institute of Ophthalmology, University College London, London, WC1E 6BT, United Kingdom*
[3]*UC Davis EyePod Small Animal Ocular Imaging Laboratory, University of California Davis, 4320 Tupper Hall, Davis, CA 95616, USA*
[4]*UC Davis Eye Center, Dept. of Ophthalmology & Vision Science, University of California Davis, Tschannen Eye Institute, 4860 Y St Suite 1E, Sacramento, CA 95817, USA*
[5]*Center for Human Ophthalmic Imaging Research (CHOIR), Dept. of Ophthalmology & Vision Science, University of California Davis, 4860 Y Street, Suite 2400, Sacramento, CA 95817, USA*
[6]*School of Engineering Science, Simon Fraser University, Burnaby, BC V5A 1S6, Canada*
[*]*guozheng.xu.23@ucl.ac.uk*

This supplement published with Optica Publishing Group on 26 September 2025 by The Authors under the terms of the Creative Commons Attribution 4.0 License in the format provided by the authors and unedited. Further distribution of this work must maintain attribution to the author(s) and the published article's title, journal citation, and DOI.

Supplement DOI: https://doi.org/10.6084/m9.figshare.30182878

Parent Article DOI: https://doi.org/10.1364/BOE.572077

Deep reinforcement learning for automatic defocus correction using OCT image intensity: supplemental document

1. Focus correction DDPG environment

The terminology of the Deep Deterministic Policy Gradient (DDPG) and their roles are explained in Table S1.

Table S1. Explanations of DDPG terminology

Terminology	Explanation
Agent	The agent is the DDPG algorithm that takes information of OCT B-scan intensities and corrects the defocus by sending signals to the tunable focus device in the system.
State	The state, or observation, is comprised of a reference B-scan and a target B-scan. The reference B-scan is an arbitrarily chosen frame that the following frames are registered to, while the target B-scan is one frame acquired after the reference B-scan affected by defocus.
Action	The action is the output of the Actor-Focus network in DDPG, which in this case is the focus correction signal to be applied to the tunable focus devices in the actual OCT system.
Policy	The policy is the weight of the Actor-Focus network, which outputs the focus correction signal in a given state of the reference and target B-scans.
Reward	The reward is a scalar value evaluating the effectiveness of the focus correction given an observation of reference and target B-scans. Details of reward definition can be found in Section 2.2.2 of the main article.

2. DDPG algorithm & hyperparameters

A detailed pseudocode description of the single-step DDPG algorithm used for OCT defocus correction is presented below:

Algorithm S1 Focus correction single-step DDPG

Randomly initialize Critic-Focus Q and Actor-Focus μ with weights
Initialize Memory with a sufficient size to accommodate training data
Define warm-up duration $\mathcal{W}$
for episode = 1, N **do**
    Acquire reference B-scan at a random focal depth
    Acquire target B-scan with random focus shift to the reference
    Average & Gaussian-filter B-scans to generate the observation (state) $\mathcal{O}$
    Acquire action $\mathcal{F}$ of focus correction signal from current policy: $\mathcal{F} = \mu(\mathcal{O})$
    for e = 1, $\mathcal{E}$ **do**
        Initialize a random focus correction noise profile $\mathcal{N}_e$ for action exploration
        Acquire reward r_e by applying focus correction signal with noise to the system
        Restore $[\mathcal{O},\mathcal{F} + \mathcal{N}_e, r_e]$ in Memory
    end for
    while episode > $\mathcal{W}$ **do**
        Sample a random batch of $\mathcal{S}$ transitions $(\mathcal{O}_i,\mathcal{F}_i,r_i)$ from the Memory
        Divide the batch into mini-batches of size $\mathcal{S}_b$
        for t = 1, $\mathcal{S}/\mathcal{S}_b$ **do**
            Update Critic-Focus by minimizing the loss:
            $$L = \frac{1}{\mathcal{S}_b}\sum_i \left(r_i - Q(\mathcal{O}_i,\mathcal{F}_i \,|\, \theta^Q)\right)^2$$
        end for

for t = 1, $\mathcal{S}/\mathcal{S}_b$ **do**
Update Actor-Focus policy using the sampled policy gradient:
$$\nabla_{\theta^\mu} J \approx \frac{1}{\mathcal{S}_b} \sum_i \nabla_{\mathcal{F}} Q\big(\mathcal{O},\mathcal{F}|\theta^Q\big)|_{\mathcal{O}=\mathcal{O}_i,\mathcal{F}=\mu(\mathcal{O}_i)} \nabla_{\theta^\mu} \mu(\mathcal{O}|\theta^\mu)|_{\mathcal{O}_i}$$
end for
end while
end for

The two tables below exhibit the DDPG hyperparameters used for *in silico* training (Table S2) and *in vivo* fine-tuning (Table S3). Hyperparameters can be further fine-tuned for better efficiency and performance to fit different system configurations.

Table S2. *In silico* model training hyperparameters

Type	Value
Training Episodes N	10,000
Warm-up Episodes $\mathcal{W}$	3,000
Noise Profiles per Episode $\mathcal{E}$	8
Critic-Focus Learning Rate	5e-4
Actor-Focus Learning Rate	5e-5
Sampled Batch Size $\mathcal{S}$	512
Mini-batch Size $\mathcal{S}_b$	64
Maximum Gaussian Noise Standard Deviation	0.4 D
Minimum Gaussian Noise Standard Deviation	0.001 D
Number of Episodes Annealing	10,000
Maximum Memory Size	10,000
#A-scan Axial Pixels	430
#B-scan Channel Division	2
State Dimensions	(430, 4)
Action Dimensions	(1,)

Table S3. *In vivo* model fine-tuning hyperparameters

Type	Value
Training Episodes N	3,200
Warm-up Episodes $\mathcal{W}$	1,500
Noise Profiles per Episode $\mathcal{E}$	5
Critic-Focus Learning Rate	7e-4
Actor-Focus Learning Rate	7e-5
Sampled Batch Size $\mathcal{S}$	512
Mini-batch Size $\mathcal{S}_b$	128
Maximum Gaussian Noise Standard Deviation	0.08 D
Minimum Gaussian Noise Standard Deviation	0.003 D
Number of Episodes Annealing	3,200
Maximum Memory Size	3,200
#B-scans acquired	21
#A-scans interpolated	400
