# Supplementary figures and images for: Deep reinforcement learning for automatic defocus correction using OCT image intensity
Source: Biomed Opt Express. 2025 Sep 26;16(10):4175–89. doi: 10.1364/BOE.572077 (PMC12532345; doi:10.1364/BOE.572077)

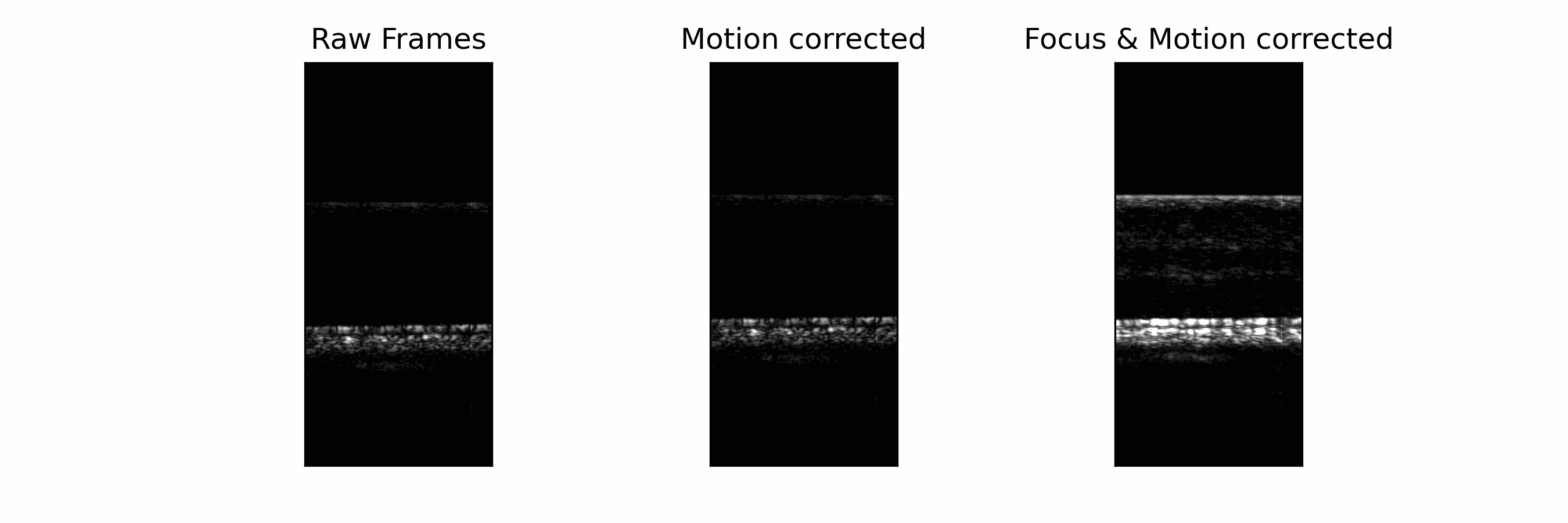

Supplement: Supplementary file 2 [file boe-16-10-4175-v001.gif]

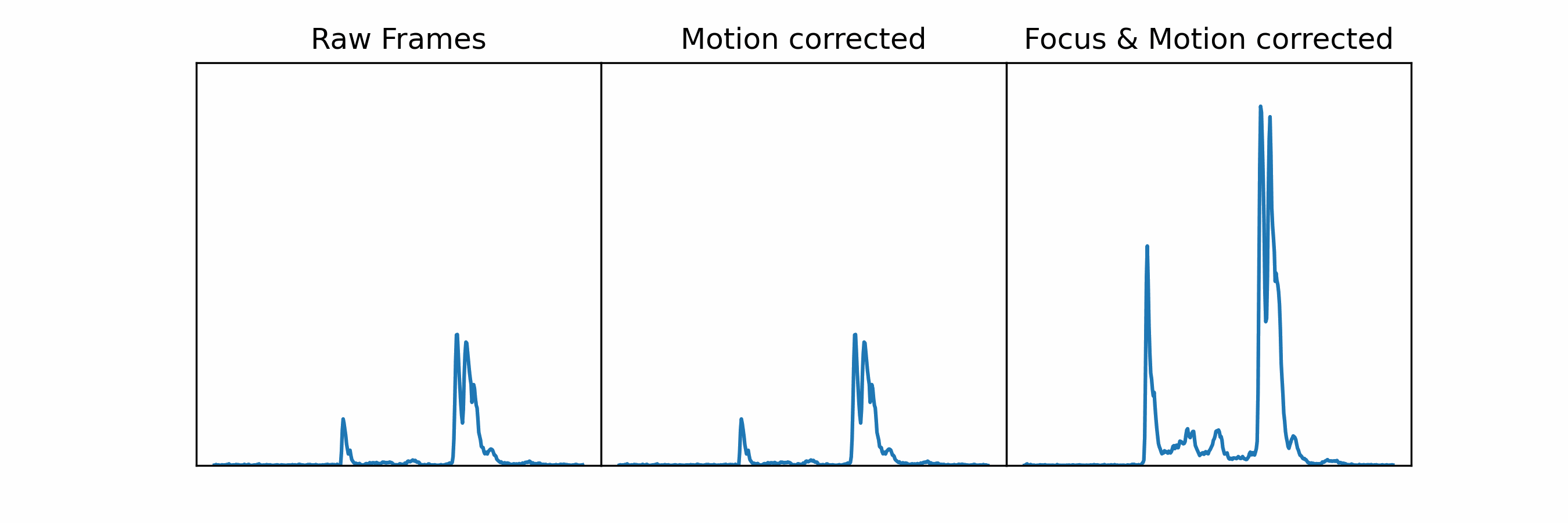

Supplement: Supplementary file 3 [file boe-16-10-4175-v002.gif]
